# Production of Mesenchymal Progenitor Cell-Derived Extracellular Vesicles in Suspension Bioreactors for Use in Articular Cartilage Repair

**DOI:** 10.1093/stcltm/szab008

**Published:** 2022-03-03

**Authors:** Jolene Phelps, Catherine Leonard, Sophia Shah, Roman Krawetz, David A Hart, Neil A Duncan, Arindom Sen

**Affiliations:** 1 Pharmaceutical Production Research Facility, Department of Chemical and Petroleum Engineering, Schulich School of Engineering, University of Calgary, Calgary, AB, Canada; 2 McCaig Institute for Bone and Joint Health, University of Calgary, Calgary, AB, Canada; 3 Biomedical Engineering Graduate Program, University of Calgary, Calgary, AB, Canada; 4 Department of Surgery, Cumming School of Medicine, University of Calgary, Calgary, AB, Canada; 5 Musculoskeletal Mechanobiology and Multiscale Mechanics Bioengineering Lab, Department of Civil Engineering, Schulich School of Engineering, University of Calgary, Calgary, AB, Canada; 6 Center for Bioengineering Research and Education, Schulich School of Engineering, University of Calgary, Calgary, AB, Canada

**Keywords:** bioprocessing, bioreactors, cartilage, chondrogenesis, extracellular vesicles, exosomes, mesenchymal stem cells, mesenchymal progenitor cells

## Abstract

Mesenchymal progenitor cells (MPCs) have shown promise initiating articular cartilage repair, with benefits largely attributed to the trophic factors they secrete. These factors can be found in the conditioned medium (CM) collected from cell cultures, and it is believed that extracellular vesicles (EVs) within this CM are at least partially responsible for MPC therapeutic efficacy. This study aimed to examine the functionality of the EV fraction of CM compared to whole CM obtained from human adipose-derived MPCs in an in vivo murine cartilage defect model. Mice treated with whole CM or the EV fraction demonstrated an enhanced cartilage repair score and type II collagen deposition at the injury site compared to saline controls. We then developed a scalable bioprocess using stirred suspension bioreactors (SSBs) to generate clinically relevant quantities of MPC-EVs. Whereas static monolayer culture systems are simple to use and readily accessible, SSBs offer increased scalability and a more homogenous environment due to constant mixing. This study evaluated the biochemical and functional properties of MPCs and their EV fractions generated in static culture versus SSBs. Functionality was assessed using in vitro MPC chondrogenesis as an outcome measure. SSBs supported increased MPC expression of cartilage-specific genes, and EV fractions derived from both static and SSB culture systems upregulated type II collagen production by MPCs. These results suggest that SSBs are an effective platform for the generation of MPC-derived EVs with the potential to induce cartilage repair.

## Graphical Abstract

**Graphical Abstract:**
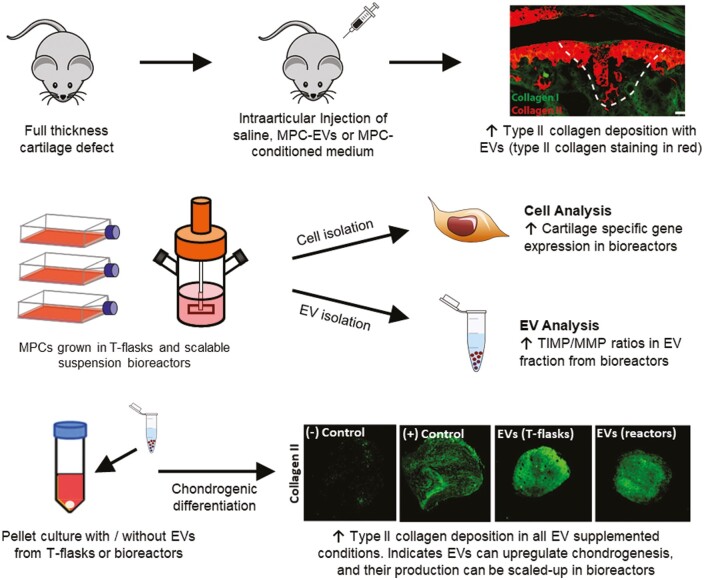
Mesenchymal progenitor cell (MPC)-derived extracellular vesicles (EVs) promote collagen II deposition when injected into the joint space of a full-thickness cartilage defect. Suspension bioreactors can be used to scale up MPC-EV production to provide clinically relevant numbers of EVs. MPC-EVs generated under the dynamic conditions found within the suspension bioreactor environment may enhance their chondrogenic characteristics.

**Significance Statement**

This work presents a scalable and clinically translatable bioprocess for the production of extracellular vesicles (EVs) from stem/progenitor cells, and the results indicate their utility in inducing cartilage repair via injection into the joint. The clinical use of EVs as compared to the cells themselves has fewer translation-related hurdles due to their nonliving nature and ease of transport/storage. The findings in this study further support the development of cartilage repair strategies using mesenchymal progenitor cell-derived EVs and highlight the importance of optimizing scalable platforms to deliver clinically relevant numbers of EVs with defined therapeutic characteristics.

## Introduction

Trauma or injury to articular cartilage can result in focal points of damage, and it is well known that these chondral defects lack the ability to spontaneously regenerate back to their original functional state, instead remaining unhealed due to the avascular and non-innervated nature of this tissue.^1^ These properties predispose cartilage injuries to undergo degenerative processes which eventually result in osteoarthritis (OA), a chronic and debilitating disease that affects more than 32.5 million individuals in the US and results in an estimated 136 billion dollar burden (including direct and indirect costs) on healthcare systems annually.^2^ Current techniques to repair damaged articular cartilage are limited, and often result in the formation of fibrocartilage, a mechanically inferior tissue that is unable to withstand physiological joint loads due to its distinct extracellular matrix (ECM) composition including a low type II to type I collagen ratio.^3^

Mesenchymal progenitor cells (MPCs) derived from a variety of tissues have demonstrated promise for the repair of cartilage defects in pre-clinical and clinical settings.^4^ A variety of treatment methods have been developed using these cells, including the stimulation of endogenous MPC populations,^5^ direct injection of MPCs into the defect site,^6-8^ assembly of MPCs into a 3D cartilage-like tissue that is then implanted into the damaged site,^9-12^ or the implantation of MPC-seeded matrices.^13,14^ Whereas implanted MPCs have been found to be able to undergo differentiation to a chondrogenic phenotype and remain viable long term in cartilage repair sites,^4^ there is increasing evidence that the therapeutic benefits observed in many studies post-transplantation may be the result of these cells regulating repair through paracrine mechanisms.^15,16^ Indeed, in a clinical study comprised of cartilage defect patients reported by de Windt et al, the co-injection of allogeneic MPCs and chondrons resulted in the complete filling of defects with hyaline-like tissue.^16^ No detectable allogeneic MPCs were found within the defect site after 1 year of implantation, thereby supporting the assertion that MPCs can exert their benefits via paracrine mechanisms. MPCs are known to produce and release biomolecules into their microenvironment which can impact the behavior of additional cell types (including immune and fibroblast populations). Over the last decade, it has been shown that many of these secreted factors are released in the form of extracellular vesicles (EVs).^17^ These membrane-bound nanovesicles, which range in size from 30 to 500nm, contain bioactive molecules, including proteins, lipids, and RNA. Upon secretion, EVs migrate throughout the local microenvironment and exert their effects on target cells through cell surface interactions and/or endocytosis. These EV-target cell interactions have been previously shown to induce endogenous repair mechanisms.^15^ It has been shown that MPC-derived EVs upregulate chondrocyte proliferation and ECM production,^18,19^ reduce inflammatory responses,^18,19^ offer chondroprotective functions in models of OA,^19-21^ and result in effective repair of osteochondral defects.^18,22,23^ Such examples illustrate that EV-based therapies may be a viable alternative to cell transplantation for the treatment of cartilage injuries and OA. The administration of EVs would be less invasive, present with fewer translational barriers, and have the potential to be more cost-effective.^15^

Defined EV populations can not be isolated directly from tissues in clinically relevant quantities. To address this limitation, in vitro methods are used to generate EVs from cultured cells. Static cell culture flasks are used extensively due to their simplicity, but this approach is not amenable to efficient scale-up, has reproducibility issues, is labor-intensive, and as a result of these limitations is not cost-effective.^24^ Conversely, stirred suspension bioreactors (SSBs) have been effectively used to scale up stem/progenitor cell production, including neural stem cells and MPCs.^25-28^ SSBs are relatively straightforward to operate, offer advantages of scalability, provide a large volume for cell growth, can support adherent cell growth with the addition of microcarriers^25-27^ or as aggregates,^29,30^ and provide a homogenous culture microenvironment due to constant mixing. However, this constant mixing does expose cells to shear in a dynamic environment that they would not be exposed to in static culture. Whereas much work has been done to optimize cell production in bioreactors, very little has been published related to the scale-up of EV production using this platform. Cell functionality is known to be impacted by the dynamic culture environment, and since EVs are produced by cells, it is reasonable to expect that EV functionality will similarly be a product of the culture environment. It is also important to keep in mind that the conditions needed to optimize cell growth may not be the same as those needed to optimize the production of EVs.

The aims of this study were to (i) test the functionality of the EV fraction in an in vivo murine cartilage defect model; (ii) evaluate MPCs and the EV fractions that they generate in static versus SSB culture conditions; and (iii) evaluate the functionality of these EV fractions using in vitro chondrogenesis as an outcome measure.

## Methods

### Cell Culture

Human adipose-derived MPCs (University of Calgary Health Research Ethics Board, ID: REB15-1005) were isolated enzymatically from abdominal subcutaneous adipose tissues (1 donor: female, age 24, BMI of 22.4, abdominoplasty). The cells were characterized previously by Jung et al as MPCs based on flow cytometry analysis (>95% expression of CD13, CD29, CD44, CD73, CD90, CD105, and CD166, and <2% expression of CD14, CD19, CD34, CD45, and HLA-DR) and confirmed adipogenic and osteogenic differentiation.^31^ Cells were serially expanded in static T-75 static tissue culture flasks (Thermo Fisher Scientific, Waltham, MA, USA). They were passaged every 72 hours (inoculated at 5000 cells/cm^2^ with 12mL of medium at each passage) in serum-free PPRF-msc6 medium at 37°C and 5% CO_2_ using standard protocols.^32^

In both static and dynamic culture for conditioned medium (CM) and EV collection, MPCs were cultured in PPRF-msc6 at 37°C and 5% CO_2_. T-75 flasks (Thermo Fisher Scientific) were used for static culture. SSBs (125mL, NDS Technologies, Vineland, NJ, USA) were inoculated with 2.3g/L Cytodex 3 microcarriers to match the surface area/volume ratios of static T-flasks (for this study, 6.25cm^2^/mL). Microcarriers were prepared as described by Roberts et al,^27^ and added to the bioreactors in 60mL of medium 4 hours prior to cell inoculation. Cells were added to the bioreactor at a density of 5000 cells/(cm^2^ of microcarrier) and then operated with 80mL of culture medium at 40rpm (corresponding to a max shear stress of 1.6 dyn/cm^2^) for 24 hours to enhance cell attachment to microcarriers. At 24 hours, the remaining 45mL of medium was added, and half of the bioreactors were switched to 80rpm (corresponding to a max shear stress of 4.1 dyn/cm^2^). These shear rates were chosen to ensure no cell damage (shown to occur at 9.76 dyn/cm^2^) or disruption and removal from the surface (shown to occur at 6.5 dyn/cm^2^).^28^ Maximum shear stress was calculated using methods published by Sen et al.^28^

Cells from passage 6 to 7 were used for CM and EV collection, as this provided enough time in culture to provide a sufficient number of cells for these studies. These cells maintained their defining MPC characteristics during the serial passaging period (unpublished data). Moreover, they maintained their clinical significance as the secretome of these cells has previously been characterized to remain consistent at these passage levels,^33^ and MPCs have been shown to not undergo significant changes in protein synthesis from passage 3 to 7.^34^ Cells from static culture were enzymatically removed using TryplE (Thermo Fisher Scientific), stained with trypan blue and counted using a hemocytometer. Cells from SSBs were sampled and the attached cell density was determined by adding crystal violet to lyse the cells and stain the nuclei.^27^ Briefly, 2×2mL samples were taken from each bioreactor and the microcarriers were washed twice with 1× Dulbecco\'s phosphate buffered saline (DPBS) and resuspended in 0.1% crystal violet with 0.1 M citric acid. After overnight incubation, the nuclei were counted using a hemocytometer. Cells/mL as determined from cell counts were converted to cells/cm^2^ by dividing by the surface area/volume ratio used in this study.

### RT-qPCR

MPCs were washed with 1× DPBS and resuspended in Trizol reagent. Total RNA was isolated using the TRIspin method,^35^ purified, quantified, and then reverse transcribed. Total RNA was isolated using the RNEasy Mini Kit (Qiagen, Hilden, Germany) with any potential contaminating DNA being digested with a RNase-Free DNase Set (Qiagen). qPCR was performed as described previously^36^ using an iCycler (Bio-Rad Laboratories, Inc., Mississauga, ON, Canada) and human-specific primers were validated as listed in Table 1. Briefly, RNA was reverse transcribed using an Omniscript RT Kit (Qiagen), and template cDNA was amplified using iQ SYBR Green Supermix (Bio-Rad Laboratories Inc.) according to the manufacturer’s reaction mix preparation and 2-step slow thermal cycling protocol. Annealing temperatures were optimized for each set of primers, and a melt curve analysis (55-95°C, 0.5°C increments for 0.05 minutes) was done for each primer pair to confirm amplicon specificity. Gene expression was normalized to 18S as this has been established as a stable housekeeping gene within our laboratory,^37-39^ and the resultant data were analyzed using the $2^{-\Delta \Delta C_{T}}$ method.

**Table 1.** Human-specific primers used for RT-qPCR (F: forward; R: reverse).

Gene	Primer sequence (5ʹ-3ʹ)	Size	°C	Eff.	Origin
18S	F: TGG TCG CTC GCT CCT CTC C	360	65	99.7	NR_003286
	R: CGC CTG CTG CCT TCC TTG G				
ACAN	F: GGT TGA GAA TGA GAC TGG AG	99	63	95.6	X80278
	R: TGC ACG ACG AGG TCC TCA CT				
HMOX1	F: ATG ACA CCA AGG ACC AGA GC	153	59	105.3	NM_002133
	R: GTG TAA GGA CCC ATC GGA GA				
MMP2	F: GTG CTG AAG GAC ACA CTA AAG AAG A	605	65	102.3	NM_004530
	R: TTG CCA TCC TTC TCA AAG TTG TAG G				
NANOG	F: TGC AGA GAA GAG TGT CGC AA	98	63	99.1	NM_024865
	R: CAT TGA GTA CAC ACA GCT GG				
SOX9	F: GTA CCC GCA CTT GCA CAA C	72	63	102.4	NM_000346
	R: TCG CTC TCG TTC AGA AGT CTC				
TGFB1	F: GGG GAA ATT GAG GGC TTT CG	388	65	105.4	NM_000660
	R: CCA GGA CCT TGC TGT ACT GC				
TIMP1	F: AAT TCC GAC CTC GTC ATC AGG	442	65	97.8	NM_003254
	R: ACT GGA AGC CCT TTT TCA GAG C				
TIMP2	F: TGA ACC ACA GGT ACC AGA TG	164	63	100.2	NM_003255
	R: GTC ACT TCT ACC GAT GCA GG				

Note: Size is specified in base pairs. °C represents annealing temperature for each primer pair; Eff. represents amplification efficiency for each primer pair (%).

### Isolation of Conditioned Medium and Extracellular Vesicles

For the cartilage defect model, the CM was obtained from MPC cultures after 72 hours. For the remaining experiments where static conditions were compared to dynamic conditions, CM was obtained from MPC cultures after 84 hours. To obtain concentrated conditioned medium (CCM), the medium was centrifuged at 2000*g* and 4°C for 10 minutes to remove pelleted cell debris and apoptotic bodies and then concentrated in Amicon Ultra-15 3kDa Filter Units (EMD Millipore, Burlington, MA, USA) by centrifuging at 4000*g* for 60 minutes. The EV fraction was isolated using differential ultracentrifugation, using a commonly reported high recovery, low specificity protocol,^40^ and it is likely to contain some contaminating proteins. To acknowledge this, we have used the term “EV fraction” throughout the manuscript. The medium was centrifuged at 2000*g* and 4°C for 10 minutes, and then the supernatant was ultracentrifuged at 105000*g* and 4°C for 2 hours (Beckman Coulter Optima L-100K, 70 Ti Rotor, 38000rpm, *k* factor = 148). A 10000g step was added for experiments comparing static to dynamic conditions to further isolate only the small EV fraction (ie, 30-150nm). For this step, after centrifugation at 2000*g*, the supernatant was further centrifuged at 10000*g* for 30 minutes to remove larger EVs prior to ultracentrifugation. The pellet containing the EV fraction was resuspended at a concentration 40-fold compared to the CM (ie, a pellet from 10mL CM was resuspended in 250 µL), in either chondrogenic differentiation medium to be used in chondrogenesis experiments, in 1× DPBS for dynamic light scattering (DLS), transmission electron microscopy (TEM), and intra-articular injection, or in RIPA buffer (1× with 10 µL/mL protease inhibitors) for biomolecular analyses. EVs were frozen at −80°C for subsequent analyses.

### Characterization of Extracellular Vesicles

EVs were characterized using a variety of methods. DLS was performed via a Zetasizer Nano Range (Malvern Panalytical, Malvern, UK) to determine the size range. Protein concentrations were obtained using Luminex MMP/TIMP and angiogenesis discovery assays (Eve Technologies, Calgary, AB, Canada). MMP (matrix metalloproteinase)-1, -2, -3, -10, -13, TIMP (tissue inhibitor of metalloproteinase)-1, -2, -3, -4, FGF-2 (fibroblast growth factor 2), HGF (hepatocyte growth factor), and VEGF-A (vascular endothelial growth factor A) were analyzed. MMPs and TIMPs are essential mediators of physiological tissue remodeling.^41^ FGF-2, HGF, and VEGF-A are recognized as classical factors secreted by MPCs^42^ and thus were analyzed to expose apparent differences in the CM and EV fractions under varying conditions. An ExoView R100 (NanoView Biosciences, Boston, MA, USA) was used for particle counts and concentration using EV-specific markers CD81, CD63, CD9, and syntenin-1, and negative marker GRP94. TEM was used to visualize the morphology and size of EVs using a Hitachi H7650 120kV microscope (Hitachi High-Tech, Tokyo, JP). Briefly, EVs were adsorbed to copper mesh grids (Electron Microscopy Sciences, Hatfield, PA, USA) for 30 minutes, washed with dH_2_O, directly stained with 2.6% uranyl acetate, and imaged at 80kV. Finally, an Exo-Check Exosome Antibody Array (System Biosciences, Palo Alto, CA, USA) was used to check for EV-specific markers. Positive EV markers FLOT1, ICAM, ALIX, CD81, CD63, EpCAM, ANXA5, and TSG101 were analyzed, as well as negative EV marker GM130 (indicative of cellular debris).

### Cartilage Defect Model

Animal studies were carried out in agreement with recommendations from the Canadian Council on Animal Care Guidelines and were approved by the University of Calgary Health Sciences Animal Care Committee.

Full-thickness cartilage defects (FTCD) were created on the tibial plateau in 12 NOD scid gamma (NOD) mice as described by Jablonski et al.^43^ Animals were administered an intraperitoneal injection of buprenorphine (0.05mg/kg) prior to surgery and anesthetized under isoflurane (Baxter) anesthesia (1.5% v/v O_2_) for the duration of the surgical procedure. Briefly, a small incision was made on the medial side of the left knee. A depth stopped 26G needle (diameter = 450 µm, length to stopper = 600 µm) was used to gently displace the patella and expose the trochlear groove of the femur. A slight pressure, combined with a twisting motion, was applied at the contact with the trochlear groove to make a circular wound that penetrated no farther than 600 µm into the underlying subchondral bone. The needle was gently removed, and the skin closed with a sterile wound clip after the FTCD was made.

The mice were grouped and intraarticularly injected 1-week post-injury with 2 µL of test material as follows: group 1: 3 mice with saline (controls), group 2: 3 mice with CCM from day 3 static (CCML, concentrated 80×), group 3: 3 mice with CCM from day 6 static (CCMH, concentrated 80×), and group 4: 3 mice with EVs from day 3 static (concentrated 320×). The 4-fold higher concentration of EVs in group 4 was to account for the 75% loss in EVs that we routinely experience due to inefficiencies associated with the standard isolation process (unpublished data). CM for each group was obtained from the same volume of medium and was treated identically. Despite significant efforts, the EV pellets obtained on day 6 consistently could not be dissociated using standard methods and were therefore excluded from this study. Immunodeficient NOD mice were used to eliminate any possibility of the host immunologically reacting to the CCM or EVs, thus making it possible to observe the effect of CCM and EVs on local cell populations directly. The mice were euthanized 4-week post-injection and then the joints were sectioned and stained.

### Chondrogenic Differentiation

MPCs (250000 cells) were pelleted in 15mL centrifuge tubes with 0.5mL of medium per pellet/tube, and the tubes were incubated at 37°C and 5% CO_2_ for 30 days. Medium changes were performed every 3 days. Human Mesenchymal Stem Cell (hMSC) Chondrogenic Differentiation (basal) Medium (Lonza, Basel, Switzerland) was used as a negative control. The addition of 10ng/mL human recombinant TGF-β3 (Lonza) served as a positive control. To evaluate the impact of EVs on chondrogenic differentiation of MPCs, EVs isolated from 5mL of CM derived from static or dynamic MPC cultures were added to each pellet in chondrogenic basal medium without TGF-β3.

### Histology and Immunofluorescence

Intact murine knee joints were dissected and fixed in neutral buffered formalin (Sigma-Aldrich) for 5 days, then decalcified in EDTA for 10 days. After decalcification was complete, samples underwent standard tissue processing for paraffin sectioning and sagittal sections (10 µm) were cut. Sections were co-stained with safranin O and fast green and graded based on a previously published scoring system by Fitzgerald et al.^44^ The parameters of the scoring system include cell morphology (0-4), matrix staining (0-3), surface regularity (0-3), thickness of cartilage (0-2), and integration with native cartilage (0-2). On this scale, uninjured native articular cartilage is 14, while the absence of cartilage is 0. Blinded grading of all images was performed by 2 independent observers. Chondrogenic cell pellets were stained with Alcian blue as previously described.^45^

Mouse knees and cell pellets were processed for immunofluorescence. Primary antibodies: type I collagen (clone 8-3A5), type II collagen (clone CIIC1), and type X collagen (clone X-AC9) (all from DSHB, University of Iowa) were directly conjugated to Dylight 488 or Dylight 500 using 10 µg Lightning Kits (Abcam). All antibodies were used at a final concentration of 0.1 µg/mL. All slides were counterstained with the nucleic acid stain (4ʹ,6-diamidino-2-phenylindole [DAPI]) (Sigma-Aldrich) and mounted using FluorSave reagent (Calbiochem, Darmstadt, Germany). Slides were imaged using a Plan-Apochromat 10× objective on an Axio Scan.Z1 Slide Scanner microscope (Carl Zeiss, Oberkochen, Germany).

The relative amounts of type I, II, and X collagen within the chondrogenic pellets (*n* = 6 per treatment group) were identified by quantifying the mean fluorescent intensity (MFI) of each channel using ImageJ (NIH) by selecting the pellet area and measuring area, integrated intensity, and mean gray value. MFI was calculated as integrated density − (area of selected cell × mean fluorescence of background readings).

### Statistical Methods

Data are presented as mean ± SD. A minimum of triplicate samples was used to evaluate conditions. One-way ANOVA followed by post hoc analysis using the Bonferroni multiple comparisons test was used to compare between conditions. The difference in means was determined to be significant if *P* < .05. GraphPad Prism was used to compute all statistics.

## Results

### The MPC EV Fraction Enhances Proteoglycan and Type II Collagen Deposition in Murine Cartilage Defects

**Figure 1.**
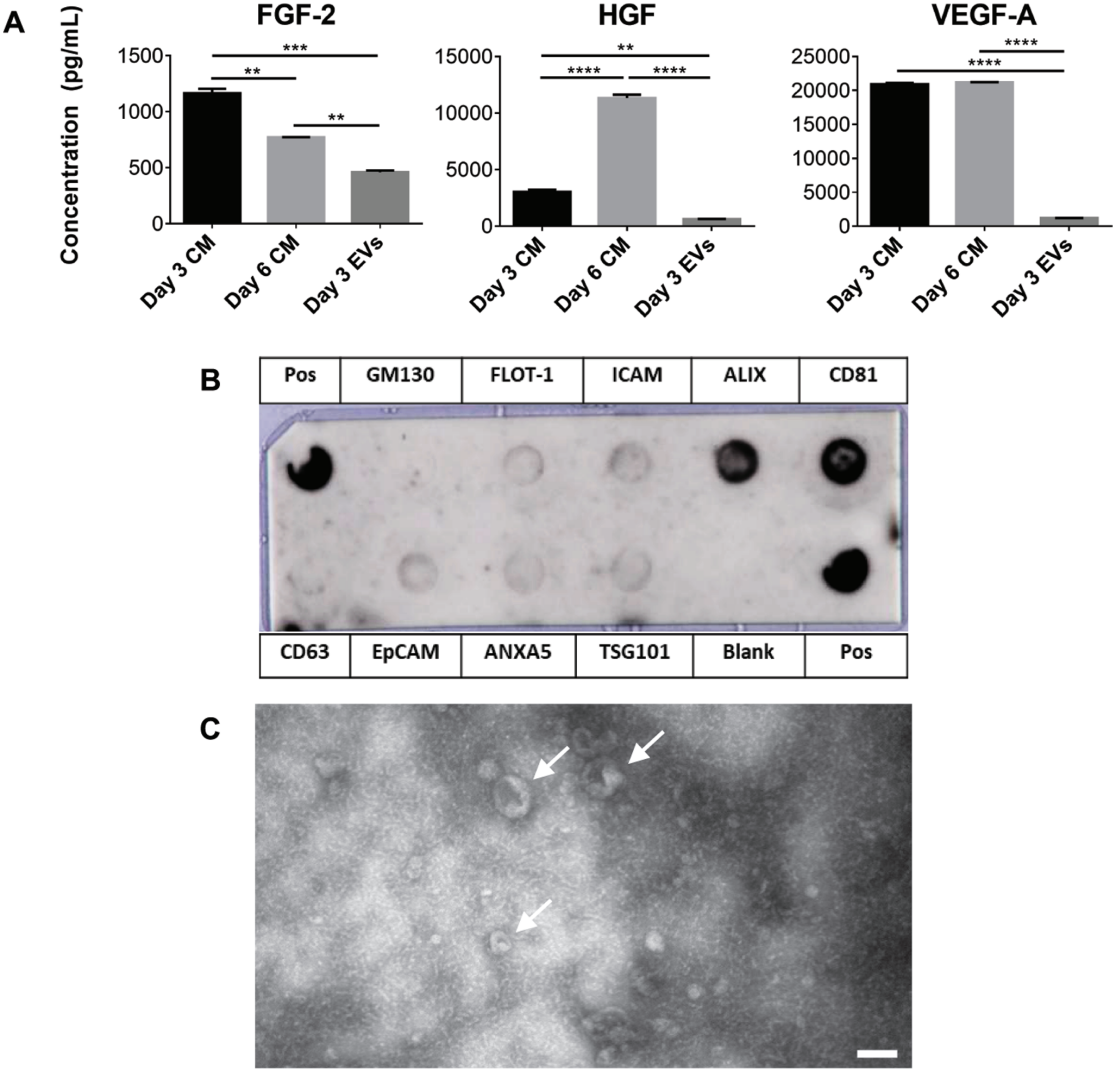
Characterization of the EV fraction and protein content compared to CM from MPCs. (A) FGF-2, HGF, and VEGF-A concentrations in day 3 and day 6 CM and in the EV fraction normalized to CM volume. (B) Antibody array for the EV fraction. (C) TEM image of the EV fraction displaying the expected cup-shape morphology (scale bar = 100nm). Arrows indicate EVs (∗∗*P* < .01, ∗∗∗*P* < .001, ∗∗∗∗*P* < .0001 relative to static controls). Abbreviations: CM, conditioned medium; EV, extracellular vesicle; FGF-2, basic fibroblast growth factor 2; HGF, hepatocyte growth factor; MPC: mesenchymal progenitor cell; TEM, transmission electron microscopy; VEGF-A, vascular endothelial growth factor A.

CM was obtained from both day 3 and day 6 MPC cultures, with a full medium change on day 3. EVs were isolated from day 3 CM. The concentrations of FGF-2, HGF, and VEGF-A in each condition were measured using Luminex. Concentrations of all 3 growth factors were significantly lower in the EV fraction compared to the CM, and there were higher levels of FGF-2 and lower levels of HGF in day 3 CM compared to day 6 CM as shown in Fig. 1A. The EV fraction was characterized to ensure it contained EVs as per the criteria defined by the International Society for Extracellular Vesicles (ISEV). Fig. 1B displays positive staining for EV-specific markers FLOT1, ICAM, ALIX, CD81, CD63, EpCAM, ANXA5, and TSG101 and negative staining for mitochondria marker GM130 which indicated that the isolated EV fraction did not contain detectable cellular debris. Minimal staining for CD63 was unexpected and could be due to low affinity of the antibody to the sample. TEM imaging, performed to visualize individual EVs (Fig. 1C), showed the typical cup-shaped morphology of EVs in the correct size range.^46^

**Figure 2.**
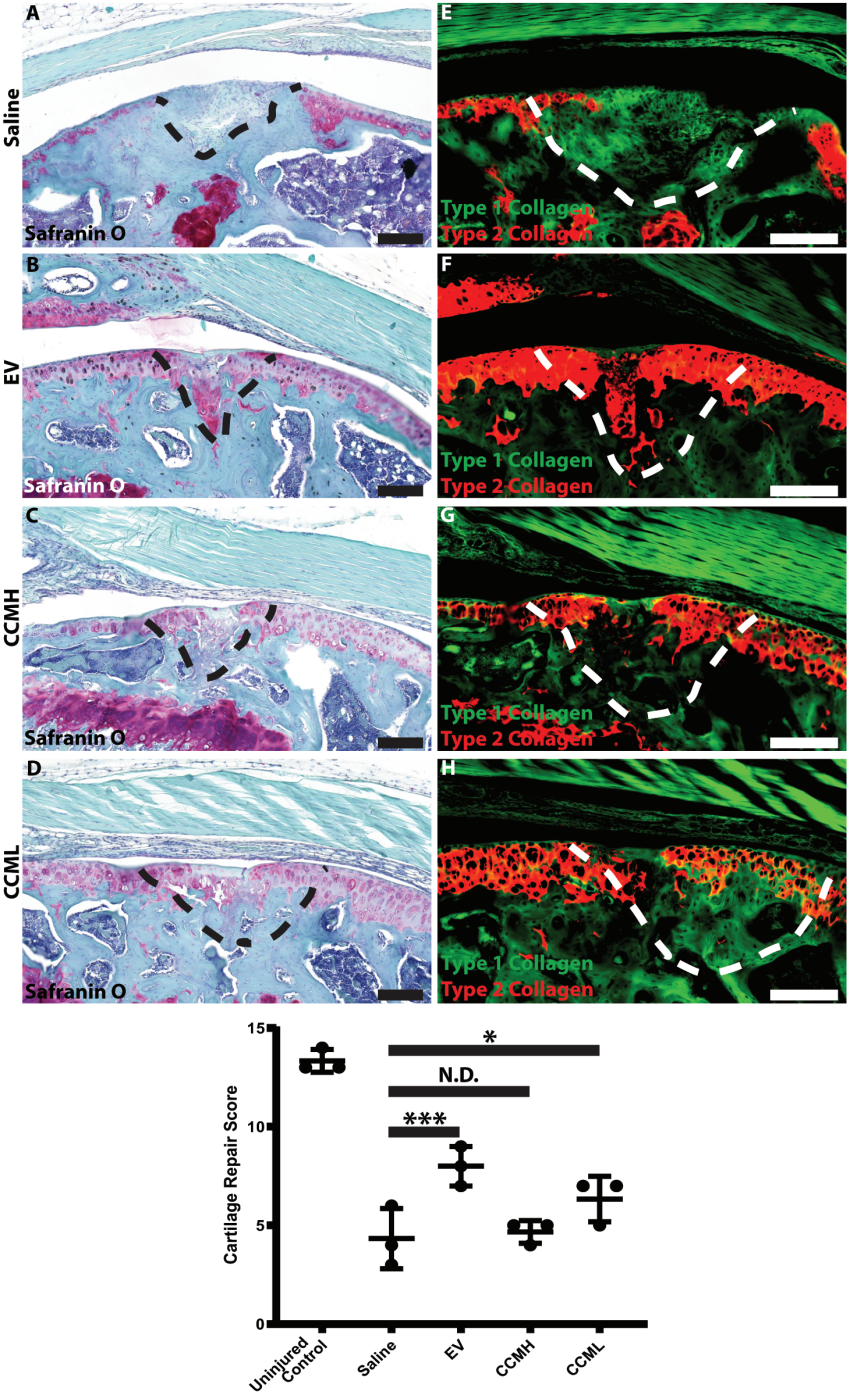
Safranin O (A-D) and type I/II collagen (E-H) staining of sectioned mouse joints. Dashed lines indicate where full-thickness defects were created. Red/purple staining for safranin O on the left indicates the presence of proteoglycans. Green and red staining on the right indicate the presence of type I and II collagen, respectively. Saline conditions showed no pronounced staining for either proteoglycans or type II collagen indicating the formation of fibrocartilage. EV and CCM conditions displayed positive staining for proteoglycans and type II collagen, with EVs providing the most prominent results (CCMH = day 6 CCM, CCML = day 3 CCM). Cartilage repair scoring showed that EV and CCML treatment groups demonstrated increased repair versus saline-treated controls, while CCMH had no effect (*n* = 3). ∗*P* < .05, ∗∗∗*P* < .001. Scale bars = 100 µM. Abbreviations: CCM, concentrated conditioned medium; EV, extracellular vesicle.

Both CCM and EVs were evaluated in a FTCD murine model. CCM was tested in addition to EVs to ensure that positive effects could be primarily attributed to the EV fraction. FTCD were induced in NOD mice within their femoral groove. One-week post-injury, the mice (*n* = 3 per group) were injected with 2 µL of either saline (group 1—controls), CCM from day 3 (group 2—CCML), CCM from day 6 (group 3—CCMH) or with EVs from day 3 (group 4). The effects of CCM and EVs were evaluated on the injured joints after 4 weeks (Fig. 2). As expected, the cartilage injury in mice treated with saline simply filled with fibrocartilage as evidenced by a lack of proteoglycan content (no red/purple staining) and only type I collagen deposition. With the injection of CCM or EVs, the defects presented with positive proteoglycan staining as well as deposition of type II collagen, suggesting, from a biochemical viewpoint, the formation of an articular-like cartilage. In particular, the animals treated with purified EVs demonstrated robust type II collagen staining within the injury site. When evaluating these treatment groups using a cartilage repair histological grading system, the EV and CCML groups demonstrated increased repair compared to the saline control group (Fig. 2); however, the CCMH group showed no significant improvement in cartilage repair versus the saline control.

### MPCs within SSBs Show Increased Chondrogenic Gene Expression, Increased EV Production on a per Cell Basis, and Reduced MMP Production in the EV Fraction

**Figure 3.**
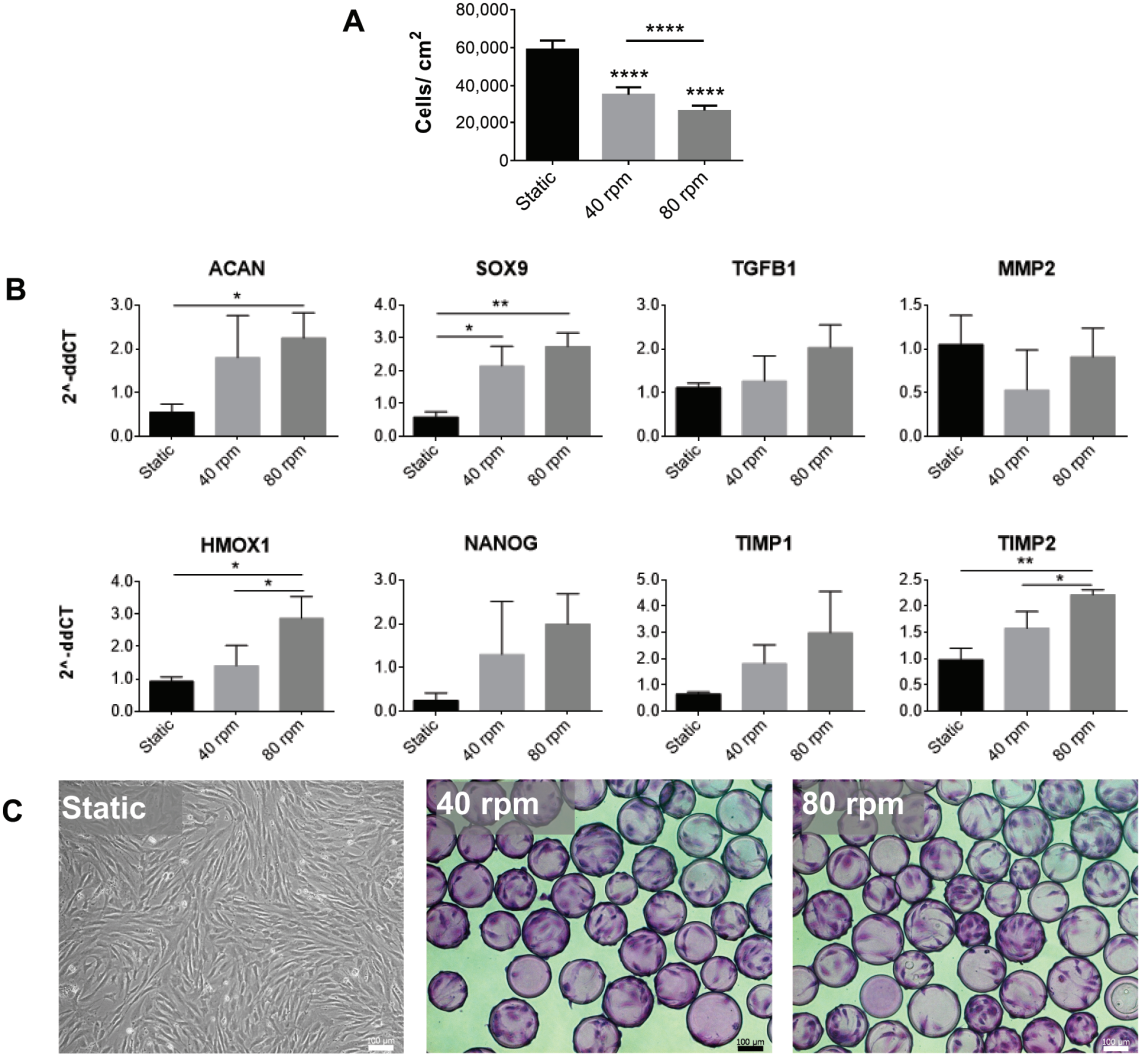
MPC cell counts, photomicrographs, and gene expression at 84 hours. (A) Cell counts at 84-hour post-inoculation. Static culture counts include only live cells as found using trypan blue exclusion, and dynamic culture counts include microcarrier-attached cells (*n* = 12). All conditions were inoculated at 5000 cells/cm^2^. (B) Relative expression of *ACAN*, *SOX9*, *TGFB1*, *VEGF-A*, *MMP2*, *HMOX1*, *NANOG*, *TIMP1*, and *TIMP2* normalized to 18S (*n* = 3). (C) Photomicrographs of cells grown in static (left) and on microcarriers in spinner flask bioreactors at 40rpm (middle) and 80rpm (right) (scale bar = 100 µm). In spinner flasks, cells were sampled and stained with crystal violet prior to imaging (∗*P* < .05, ∗∗*P* < .01, ∗∗∗∗*P* <.0001 relative to static controls). Abbreviations: ACAN, aggrecan; HMOX1, heme oxygenase 1; MMP, matrix metalloproteinase; MPC, mesenchymal progenitor cell; SOX9, SRY-box transcription factor 9; TGFB1, transforming growth factor beta 1; TIMP, tissue inhibitor to metalloproteinase.

To produce clinically relevant numbers of EVs for cartilage repair, a scalable bioprocess using SSBs was undertaken. Under the differing conditions, growth and gene expression of the cells, the amount of EVs, and protein content present in the EV fractions were evaluated. MPCs were inoculated at 5000 cells/cm^2^ into static T-flasks, as well as suspension bioreactors at two speeds: 40rpm with a maximum shear stress of 1.6 dyn/cm^2^ and 80rpm with a maximum shear stress of 4.1 dyn/cm^2^. Cytodex 3 microcarriers were used in the bioreactors to enable cell attachment and were added at an amount to keep the surface area/medium volume constant across both static and dynamic conditions. Cell density as measured at the time of harvest (84 hours) is presented in Fig. 3A and visualized in Fig. 3C. Dynamic conditions within SSBs led to reduced cell proliferation, with higher shear corresponding to lower final cell numbers. However, upregulated expression of *ACAN*, *SOX9*, *TGFB1*, *VEGF*, *HMOX1*, *NANOG*, and *TIMP2* (Fig. 3B) was also observed under SSB conditions, while *MMP2* levels remained relatively consistent, and no change in *TIMP1* expression was observed.

**Figure 4.**
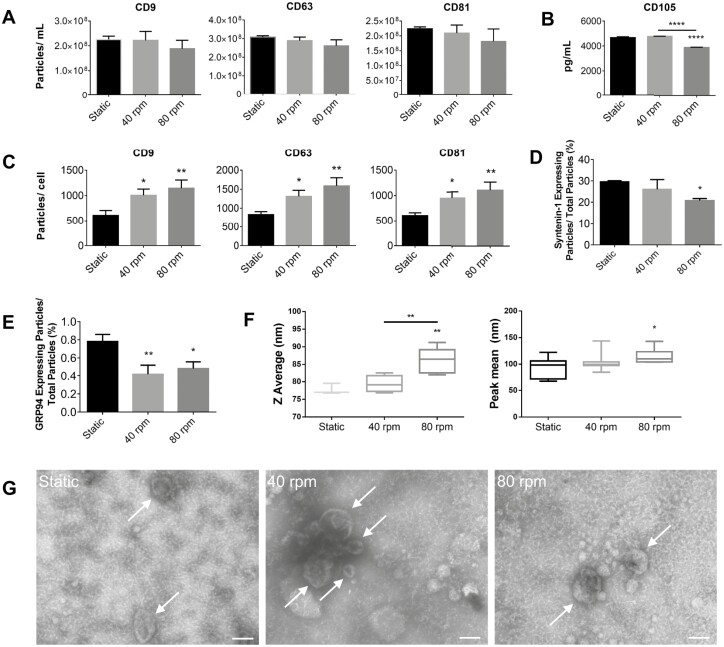
(A) ExoView particle counts for CD9, CD63, and CD81 expressing particles (*n* = 3). (B) CD105 concentration as measured by Luminex (*n* = 3). (C) CD9, CD63, and CD81 positive particles per cell (*n* = 3). (D) Syntenin-1-expressing particles measured as a percentage of total particles. (E) GRP94-expressing particles measured as a percentage of total particles. (F) DLS intensity-based *Z* average and peak mean particle diameters (*n* = 3). *Z* average is the cumulant average which assumes a monodisperse population and is highly influenced by large, aggregated particles as well as smaller peaks. Peak mean takes the average of an individual peak and is less influenced by outliers but may not account for all data. It is important to note that DLS is strongly influenced by larger particles as they scatter more light. (G) TEM images of EV fractions displaying the expected cup-shape morphology (scale bar = 100nm). Arrows indicate EVs (∗*P* < .05, ∗∗*P* < .01, ∗∗∗∗*P* < .0001 relative to static controls). Abbreviations: DLS, dynamic light scattering; EV, extracellular vesicle; TEM, transmission electron microscopy.

EVs were obtained from the CM of static and SSB MPC cultures at 84 hours, isolated via ultracentrifugation, and the pellets were resuspended in DPBS. CD9-, CD63-, and CD81-positive particle counts (ie, EV counts) were obtained, exhibiting a slight reduction with increasing shear (Fig. 4A). CD105 concentrations were determined via Luminex with a lower concentration found in the 80rpm condition. The volume of medium was consistent among all conditions, whereas the cell number was not consistent as shown in Fig. 3A. EV yield (EV/cell) was found to significantly increase at higher shear rates (Fig. 4B). Average particle diameter as measured by DLS is shown in Fig. 4F. Larger particles were detected under 80rpm conditions. ExoView cargo analysis was undertaken to evaluate the expression of the internal marker syntenin-1, expressed as a percentage of syntenin-1 expressing particles/total particles (Fig. 4D), as well as the negative marker GRP94 (Fig. 4E). Syntenin-1 was expressed in 30% of particles in static conditions down to 20% in 80rpm dynamic conditions, while GRP94 was hardly detectable at <1% of particles, as expected. TEM imaging illustrated typical cup-shaped morphology for all conditions (Fig. 4G).

**Figure 5.**
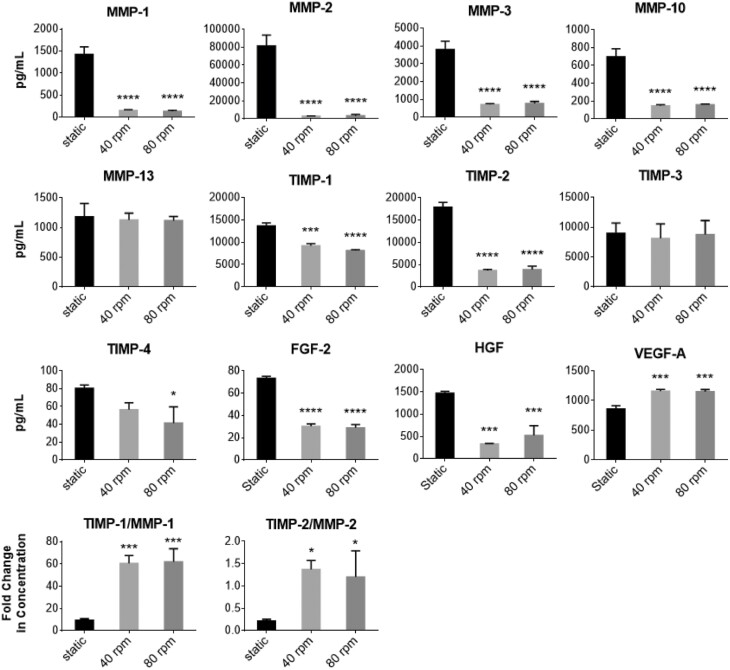
Protein concentrations in the EV fractions of static and dynamic conditions MPC cultures as measured by Luminex. *N* = 3 for all conditions. All samples were concentrated at a factor of 40×, the volume of the medium from which they were isolated (∗*P* < .05, ∗∗∗*P* < .001, ∗∗∗∗*P* < .0001 relative to static controls). Abbreviations: EV, extracellular vesicle; FGF-2, basic fibroblast growth factor 2; HGF, hepatocyte growth factor; MMP, matrix metalloproteinase; MPC, mesenchymal progenitor cell; TIMP, tissue inhibitor of metalloproteinase; VEGF-A, vascular endothelial growth factor A.

Luminex analyses for several MMPs and TIMPs, as well as FGF-2, HGF, and VEGF-A present within the EV fraction are presented in Fig. 5. A significant reduction in MMP-1, -2, -3, and -10 was observed in SSBs as well as a reduction in TIMP-1, TIMP-2, and TIMP-4. Taking the ratio of TIMP/MMP, SSB conditions produced higher concentrations of TIMP per MMP in the EV fraction. Lower concentrations of FGF-2 and HGF, and higher concentrations of VEGF-A were seen in the EV fraction from the SSB conditions. No significant differences were found in EV fractions obtained from the 40 and 80rpm conditions, apart from TIMP-1, which was slightly lower at 80rpm. Despite small differences in the protein content of the EV populations, larger differences were detected in MPC gene expression between the conditions, with the largest differences occurring between the static and 80rpm conditions. Therefore, for testing the functionality of static compared to SSB conditions, only those isolated from high shear (80rpm) conditions were used for further study.

### EVs from Both Static and SSB Culture Induce Type II Collagen Deposition During Chondrogenesis of MPCs

**Figure 6.**
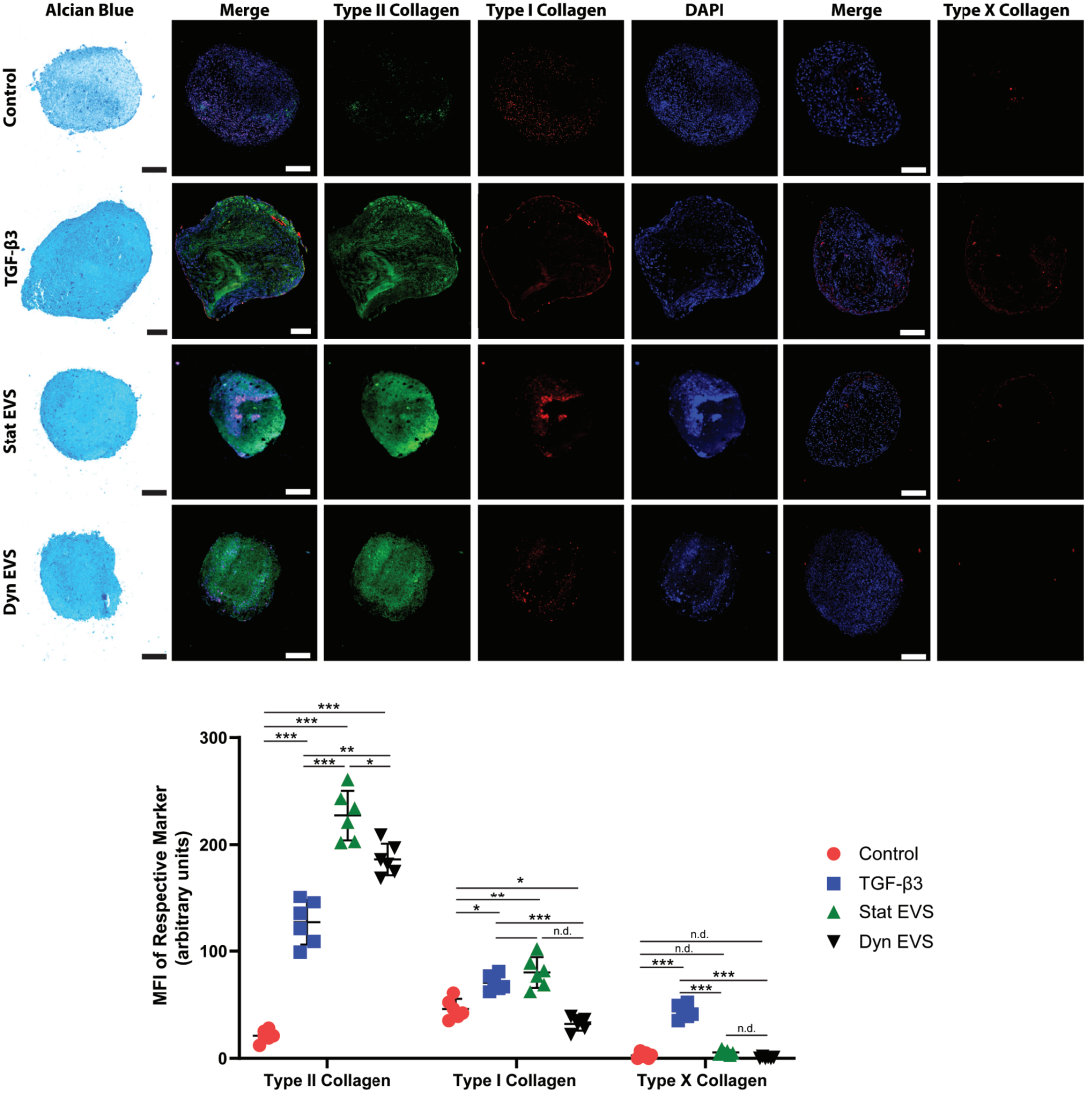
Alcian blue, type I/II/X collagen, and DAPI staining for chondrogenic pellets. 250000 MPCs were pelleted in each condition on day 0. The control condition contained chondrogenic basal medium without growth factor supplementation (negative control). The control + TGF-β3 condition contained control medium with the addition of 10ng/mL TGF-β3 (positive control). Static (Stat) and SSB (Dyn) conditions contained negative control medium with the addition of EVs from static and SSB (80rpm) culture conditions. Pellets were fixed after 30 days of culture, sectioned, and stained accordingly. Pellet quality scoring was identified by quantifying the MFI of each channel (*n* = 6). Scale bars = 25 µM. Abbreviations: DAPI, 4ʹ,6-diamidino-2-phenylindole; EVs, extracellular vesicles; MFI, mean fluorescent intensity; MPCs, mesenchymal progenitor cells; SSB, stirred suspension bioreactor; TGF, transforming growth factor.

To evaluate the functionality of the EVs generated under the two culture conditions, EVs were tested in an in vitro model of chondrogenesis. MPC chondrogenesis was used as an in vitro model to test if the EVs could induce collagen II production similar to what was seen in vivo. Although MPC benefits in cartilage repair may not be inherently due to MPC differentiation into cartilage-like tissue, their ability to differentiate does lend to a functional model for testing chondrogenesis. MPCs (250000 cells) were pelleted in standard chondrogenic basal medium and incubated for 30 days with periodic medium changes. The cell pellets were subsequently sectioned and stained with Alcian blue to visualize proteoglycans, and immunostained for type I collagen, type II collagen, type X collagen, and DAPI as shown in Fig. 6. Negative controls without the addition of growth factors contained minimal amounts of proteoglycans or types I/II collagen. Positive controls treated with TGF-β3 presented with robust staining for proteoglycans and type II collagen, while cell pellets treated with EVs from both static and SSB (80rpm) cultures displayed greater proteoglycan and type II collagen staining than the TGF-β3 group. All groups examined displayed minimal staining for type I collagen, with the EVs from static culture showing the least type I collagen expression. While little to no type X collagen staining was observed, the TGF-β3 group presented with more staining than any other group.

## Discussion

While stem/progenitor cell treatments for cartilage injury and/or OA have shown promise, it is still common to observe the development of fibrocartilage post-implantation in an injury site which, relative to native articular cartilage, has a compromised capacity to withstand normal biomechanical forces within a joint over the long term.^1^ There is also increasing evidence that the life span of MPCs following transplantation is shorter than previously thought, and that bioactive factors secreted by the implanted cells while they were viable may be responsible for many of the subsequently observed tissue regeneration related improvements.^1^ These bioactive factors can be found within the CM of MPC cultures, and published studies have demonstrated the benefits of CM for a variety of disease models, including for cartilage repair and OA.^1,47,48^ To identify the factors in the CM responsible for tissue regeneration, fractionations have been undertaken, and it has been shown that the fraction containing EVs accounts for the majority of the benefits provided by MPC-CM, with their effects after administration being similar to those reported following cell implantation.^21^ The inability to predict cell behavior after implantation is one of the several impediments to securing regulatory approval for the widespread use of MPCs as a therapeutic option; and the production and use of EVs overcomes many of these regulatory hurdles.^49^ In fact, the use of EVs in cell-free therapies offers several advantages from a clinical perspective: (i) signals can be bioengineered and scaled to specific dosages, (ii) their nonliving nature enables them to be more efficiently stored and transported, and (iii) they are considered safer as they cannot replicate and are less likely to trigger an immunogenic response.^49, 50^ Moreover, the minimally invasive manner in which EVs can be administered (eg, via syringe or catheter) significantly decreases or eliminates the need for traumatic surgical intervention, and thus, has greater potential for widespread adoption, and significantly reduced healthcare costs.

In the present study, we demonstrated the potential for MPC-EVs to repair cartilage injuries, including significant type II collagen deposition in a FTCD model. The response effects using the EV fraction as compared to complete CM were larger despite the higher overall protein content present in unfractionated CM. This could have been due to increased stability of bioactive factors when bound to the bilayer membrane of EVs, improved presentation of bioactive factors to target cells, or the selective targeting mechanisms associated with EVs.^51,52^ To our knowledge, no other studies to date have evaluated the effect of MPC-EVs specifically in FTCDs. Zhang et al^22^ demonstrated that MPC-EVs could promote complete restoration of cartilage in osteochondral bone defects in rats, with control groups presenting with fibrocartilage-like tissue. A study by Wang et al^53^ looked at the effects of chondrogenic progenitor cell-derived EVs and similarly showed effective articular cartilage repair. However, MPCs may be considered more clinically applicable as they can be obtained from patients with minimal invasiveness in comparison to cells harvested from cartilage tissue that require an additional surgery.^1^ Moreover, the expansion of chondrocytes in culture, a key step required to generate clinically relevant quantities of EVs, typically results in their rapid dedifferentiation.^54^ A significant challenge in translating MPC-EVs to the clinic is the large number of EVs required per dose, as well as the cell population heterogeneity associated with traditional static culture methods, which can result in a lack of consistency in EV functionality.^55^ Phinney et al^49^ indicated that the treatment of a range of diseases in rats would require ~50-250 µg of EVs to be administered directly to the site of injury, with the estimation that 80 µg of EVs can be collected in 48 hours from 2 million MSCs (under static conditions). Microcarrier culture in controlled bioreactors has been shown to support the expansion of MPCs,^25,26^ and indeed has been found to result in less heterogeneity between MPC populations from different donors.^56^ This suggests that microcarrier culture may also result in a lower degree of heterogeneity between EV populations derived from different MPC donors, since secreted EV population characteristics are reflective of the parent cells from which they are collected.^52^ Whereas this has not yet been definitively reported, evaluating differences in therapeutic efficacy between donor MPC-EV populations under both static and SSB conditions would be of significant relevance to the field. The current study has demonstrated that SSBs, known to be a scalable cell production platform^57^ due to their ability to maintain a controlled environment and hold culture volumes greater than practically possible in static culture vessels, can also serve as a means to generate large numbers of EVs. Other bioreactor platforms that have been shown to support enhanced production of EVs are vertical wheel bioreactors^58^ and scaffold perfusion bioreactors.^59^

The benefits of shear on MPC chondrogenesis have been reported in several studies.^60-62^ In combination with compressive forces, shear was shown to significantly upregulate chondrogenic gene expression as well as glycosaminoglycan and type II collagen deposition.^60,61^ Furthermore, when MPCs were cultured in a scaffold in a flow-perfusion bioreactor which exposes the cells to fluid shear, ECM deposition and type II collagen production was enhanced compared to static controls.^62^ In this study, the dynamic conditions in SSBs upregulated the gene expressions of *ACAN* and *SOX9*, indicating that shear may enhance the chondrogenic potential of MPCs. It also upregulated the expression of *HMOX1*, *NANOG*, *TGFB1*, *TIMP1*, and *TIMP2*. The overexpression of *HMOX1* in MPCs has been shown to have a positive effect in improving OA-related symptoms. For example, lipopolysaccharide-treated chondrocytes (which mimic the inflammation-impacted chondrocytes associated with OA) exhibited lower pro-inflammatory factors and increased expression of type II collagen and aggrecan when co-cultured with *HMOX1* expressing MPCs.^63^ In addition, when the MPCs were implanted into a surgically induced canine OA model, an improvement in limb function and a reduction in pain were seen.^63^ Interestingly, the overexpression of the pluripotency marker *NANOG* in MPCs has also been shown to improve chondrogenesis,^64^ and when induced in chondrocytes, maintains their phenotype and function in vitro.^65^

MMPs are enzymes that break down ECM proteins, whereas TIMPs act to inhibit the effects of MMPs. The ratio or balance between MMPs and TIMPs is therefore important in maintaining proper tissue remodeling, and low TIMP/MMP ratios have been reported to be a large contributing factor to OA development.^66^ Furthermore, overexpression of MMPs is indicative of OA activity,^42^ and increased TIMP expression increases anabolic activity in the injury response.^67^ Notably, TIMP/MMP ratios were found to be significantly higher for SSB cultures. MMP concentrations were significantly lower in EV fractions obtained from SSB cultures, although TIMP-1, -2, and -4 concentrations were also surprisingly lower, despite the increased expression of *TIMP1* and *TIMP2* in the MPCs. The reduction of MMP concentration and higher TIMP/MMP ratios found in the EV fraction obtained from dynamic conditions could be indicative of enhanced protective properties and increased anabolic activity in cartilage repair applications. Furthermore, FGF-2 and HGF concentrations within the EV fraction were lower in SSB conditions while there was a higher concentration of VEGF-A. While HGF has been correlated with facilitating cartilage repair,^68^ both FGF-2 and VEGF-A have been associated with degenerative or catabolic effects on cartilage.^69,70^ Although FGF-2 is present within PPRF-msc6, FGF-2 within culture medium has been reported to deplete after as little as 24 hours in culture.^71^ These findings not only have implications in cartilage repair, but provide more evidence for the need for further understanding the effect culture conditions can exert on the therapeutic potential of MPCs and their secretome.

EV cargo is known to be reflective of both the physical and functional nature of the parent cell including its pathophysiological state, as well as environmental factors.^72^ For example, there is evidence to suggest that EVs obtained during chondrogenic induction of MPCs may be more efficient for cartilage regeneration than EVs derived during non-chondrogenic culture, at least in part due to upregulated expression of miR-320c.^73^ Given that the MPCs produced in SSBs in the current study had characteristics indicative of chondrogenesis to a greater extent than observed from static culture conditions, the EV populations that they produce would be expected to have increased efficacy for cartilage repair. Certainly, cells exposed to higher shear levels in SSBs produced more EVs on a per cell basis than did their static culture counterparts. Whereas cell confluence has been reported to be negatively correlated to EV secretion rates,^74^ the higher EV yields under SSB conditions is consistent with other studies comparing static and dynamic cultivation techniques.^59,75^ In addition, the average size of EVs was found to increase at higher shear rates. EV populations are made up of subsets of vesicles that include exosomes (30-150nm); formed by the inward budding of late endosomes) and microvesicles (100-1000nm; shed from the plasma membrane).^76^ The observed increase in average EV size under shear conditions may reflect a shift in the ratio of exosomes to microvesicles. Cell-derived vesicles in the range of 100-150nm have been proposed as having chondrogenic capabilities.^53^

Thus, whereas reduced MPC expansion was observed in SSBs relative to static culture, the nature of the resulting cell populations may make them more appropriate for the production of EVs for cartilage-related applications. The scalable nature of the bioreactor platform can more efficiently facilitate the production of large cell numbers, and in a better-controlled culture environment than what can be achieved in static culture. This supports the use of SSBs as compared to static culture methods for the production of clinically relevant quantities of MPC-derived EVs with characteristics that make them more suitable for cartilage repair.

We tested EV fractions isolated from both static and SSB conditions on pellet cultures of MPCs to determine if these biological nanoparticles could induce chondrogenesis, similar to what is observed in vivo. Indeed, the addition of MPC-EVs induced significant type II collagen deposition. While MPC-EV supplementation has not been reported previously as a mechanism for enhancing chondrogenesis in MPC pellet culture, articular chondrocyte-derived EVs have been described in the literature as providing similar results regarding enhanced type II collagen production during MPC chondrogenesis, promoting proliferation and differentiation of MPCs without TGF-β supplementation.^77^ Such findings demonstrate that MPC-EVs may contain signaling factors that can induce MPC differentiation and thus, may be one mechanism by which they exert their therapeutic benefits during cartilage repair.

In the future, there would be merit in using a more thorough and less forceful purification process to isolate EVs. Although current methods may improve EV purification, they suffer from significantly reduced yield and increased cost. The inability of current protocols to isolate pure EV populations with high yield and efficiency while simultaneously removing contaminants is a key driving force behind the extensive efforts currently underway to develop efficient EV separation methods that can serve as a standard in the field.^78^ Specific to ultracentrifugation, many protocols do use a second ultracentrifuge run in an attempt to wash out contaminating proteins from the CM, although it is known that a significant proportion of the contaminating proteins tend to remain even after this procedure.^79^ In fact, data from Webber and Clayton^79^ demonstrated significant reductions in EV yield while contaminating proteins remained at approximately the same ratio to the EVs. As mentioned in the methods, day 6 EV pellets could not be dissociated as they were very sticky and aggregated, thus could not be evenly resuspended for thorough analysis (even with the addition of EDTA). This is a phenomenon that has also been seen for highly confluent cell cultures, or when the cells have reached their plateau phase, due to the actions of extracellular DNA lost from cells.^80^ While the addition of DNase could be helpful, there is a concern in its use preceding functional studies. The presence of DNase may provide a protective effect in in vivo models,^81^ which could be beneficial in clinical applications, but not, however, for studies aimed at discovering the direct effects of EVs. Furthermore, a study by Torralba et al^82^ demonstrated that DNase treatment significantly reduced the ability of EVs to trigger activation of the desired outcome in recipient cells. There is a high need for a purification process that can isolate EVs efficiently while retaining their surface properties, preventing aggregation, and removing contaminants. As the production of fibrocartilage is a significant problem associated with many currently available cartilage repair options,^1^ either the use of EVs on their own, or combining EVs with other treatments could potentially improve type II collagen deposition and lead to the generation of more robust articular cartilage regeneration. There is still a significant need to evaluate optimal dosing and time of treatment in clinical models, to define appropriate culture conditions (ie, oxygen tension, culture medium, period, and time of harvest) for the production of MPC-EVs, and to determine the specific mechanism by which they act. It is clear that there is a level of correlation between culture conditions and the composition, and likely the functionality of the corresponding EVs. With this emerging EV field still in its infancy, the present studies illustrate that robust systems with well-defined culture conditions can lead to enhanced understanding of the regulation of EV production and further the potential of EVs in achieving clinical relevance.

## Conclusion

MPC-EVs have shown promise as an alternative to cell implantation for the regeneration of damaged cartilage. However, a clear need remains for the development of scalable bioprocesses to produce clinically relevant doses of appropriately functional MPC-EVs. In particular, dynamic processes where MPCs are exposed to shear may be more beneficial for cartilage repair applications. While the mechanism(s) of action of MPC-EVs are still largely unknown, the induction of type II collagen deposition in both the in vitro and in vivo models studied in the present research offers promise to the field. These findings support further development and optimization of bioprocesses and treatment strategies involving MPC-EVs for the repair of articular cartilage.

## Data Availability

All data generated or analyzed in this study are included in this manuscript.
